# The Role of the Interplay Between Autophagy and NLRP3 Inflammasome in Metabolic Disorders

**DOI:** 10.3389/fcell.2021.634118

**Published:** 2021-03-16

**Authors:** Shuangyu Lv, Honggang Wang, Xiaotian Li

**Affiliations:** Institute of Biomedical Informatics, Bioinformatics Center, School of Basic Medical Sciences, Henan University, Kaifeng, China

**Keywords:** autophagy, NLRP3 inflammasome, glucose metabolic disorders, uric acid metabolic disorders, inflammation-related metabolic disorders

## Abstract

Autophagy is an important and conserved cellular pathway in which cells transmit cytoplasmic contents to lysosomes for degradation. It plays an important role in maintaining the balance of cell composition synthesis, decomposition and reuse, and participates in a variety of physiological and pathological processes. The nucleotide-binding oligomerization domain-like receptor family, pyrin domain-containing 3 (NLRP3) inflammasome can induce the maturation and secretion of Interleukin-1 beta (IL-1β) and IL-18 by activating caspase-1. It is involved in many diseases. In recent years, the interplay between autophagy and NLRP3 inflammasome has been reported to contribute to many diseases including metabolic disorders related diseases. In this review, we summarized the recent studies on the interplay between autophagy and NLRP3 inflammasome in metabolic disorders to provide ideas for the relevant basic research in the future.

## Introduction

Autophagy is a closely coordinated process that isolates proteins and damaged or aged organelles in double membrane vesicles called autophagosomes which eventually fuse with lysosomes, leading to the degradation of isolated components (Behrends et al., 2010). It plays an important role in maintaining the balance of cell synthesis, decomposition and reuse (Wei et al., 2016). Abnormal autophagy is involved in the development of the pathological processes such as cardiomyopathy (Mclendon et al., 2014), neurodegenerative diseases (Mclendon et al., 2014), type II diabetes (Sarparanta et al., 2017), and cancer (Amaravadi et al., 2011). The nucleotide-binding oligomerization domain-like receptor family, pyrin domain-containing 3 (NLRP3) inflammasome can induce the maturation and secretion of interleukin-1 beta (IL-1β) and IL-18 by activating caspase-1 (Schroder and Tschopp, 2010; Strowig et al., 2012). Studies have shown that the abnormal activation of NLRP3 inflammasome contributes to many diseases including type 2 diabetes, atherosclerosis, and steatohepatitis (Mridha et al., 2017; Van Der Heijden et al., 2017; Zhai et al., 2018). Recently, more and more studies have shown that there is an interplay between autophagy and NLRP3 inflammasome in macrophage and the interplay plays an important role in many diseases including metabolic diseases, the mechanism of which remains to be elucidated (Biasizzo and Kopitar-Jerala, 2020). In this review, the interplay between autophagy and the NLRP3 inflammasome and its mechanism are explored in metabolic disorders to provide ideas for the relevant basic research in the future.

## Overview of Autophagy

**FIGURE 1:**
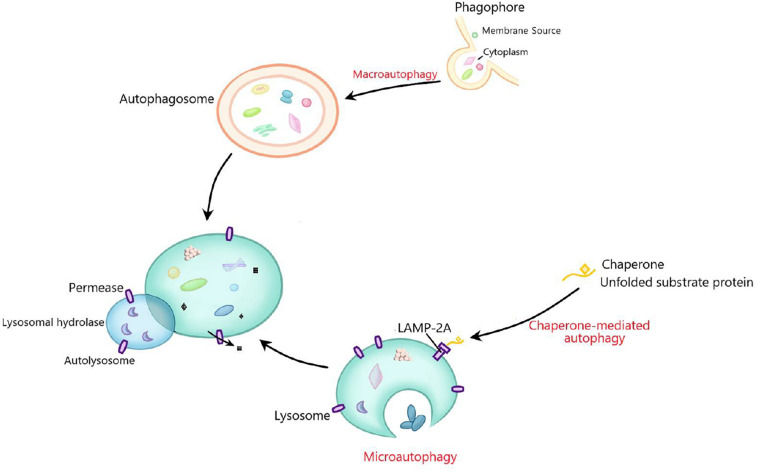
The general process of macroautophagy, microautophagy, and chaperone-mediated autophagy. Macroautophagy mainly degrades organelles and microorganisms. The degraded substances are first encapsulated by double membrane vesicles to form autophagosomes, and then fuse with lysosomes for degradation. Microautophagy does not form autophagosomes, but mainly degrades cell components by directly engulfing the cytoplasm at the lysosomal membrane through invagination and or septation. Chaperone-mediated autophagy is a selective autophagy process in which degraded proteins are transported to lysosomal chambers after binding with chaperones, and then digested by lysosomal enzymes.

Autophagy is a conserved process of self-sustaining internal environment stability, in which abnormal proteins and organelles are encapsulated by the bilayer membranes and transported to lysosomes for subsequent degradation (Sir et al., 2010; Qiu et al., 2014). Autophagy can be categorized as microautophagy, macroautophagy and chaperone-mediated autophagy according to the inducing signal, action time, target type, and transport pathway into lysosomes (Feng et al., 2014; Gomes et al., 2017). Macroautophagy, the most studied autophagy, is mainly responsible for the degradation of organelles and microorganisms. In this process, the degraded substance is encapsulated by a double membrane vesicle to form autophagosome, which then fuses with lysosome for degradation (Cheon et al., 2019). Microautophagy, without forming autophagosome, mainly degrades cell components by directly engulfing the cytoplasm at the lysosomal membrane through invagination and or septation (Oku and Sakai, 2018). Chaperone-mediated autophagy is a selective autophagy process in which intracellular proteins are transported to lysosomal chambers after binding with chaperones, then digested by lysosomal enzymes (Kaushik and Cuervo, 2012; Figure 1). Moreover, mitochondrial autophagy, also called mitophagy, is a special kind of autophagy, which selectively degrades organelle mitochondria and is largely responsible for mitochondrial quality control (Ashrafi and Schwarz, 2013; Ney, 2015). There are many factors affecting autophagy, such as Ca^2+^ concentration, immune or inflammatory stimulation, endoplasmic reticulum stress, nutritional deficiency, and accumulation of damaged cells or organelles (Tooze and Yoshimori, 2010; Mizushima et al., 2011). Under physiological conditions, autophagy often occurs at the basic maintenance level. When the body is in a pathological state, significantly enhanced autophagy can clear the abnormal proteins in cells, which is conducive to cell survival (Glick et al., 2010). The effect of autophagy on cells is a “double-edged sword,” because if autophagy is maintained at a high level, autophagy will lead to cell death (Liu and Levine, 2015; Garcia-Huerta et al., 2016). Autophagy plays a key role in many basic physiological processes, including development, protein quality control, innate immunity and cell survival. It is well known that abnormal autophagy contributes to the occurrence of many diseases, including cancer, liver diseases, neurodegenerative diseases, type-II diabetes, and inflammation (Debnath, 2011; Murrow and Debnath, 2013). So far, the mechanism of autophagy in diseases is not fully clear.

## Overview of NLRP3 Inflammasome

**FIGURE 2:**
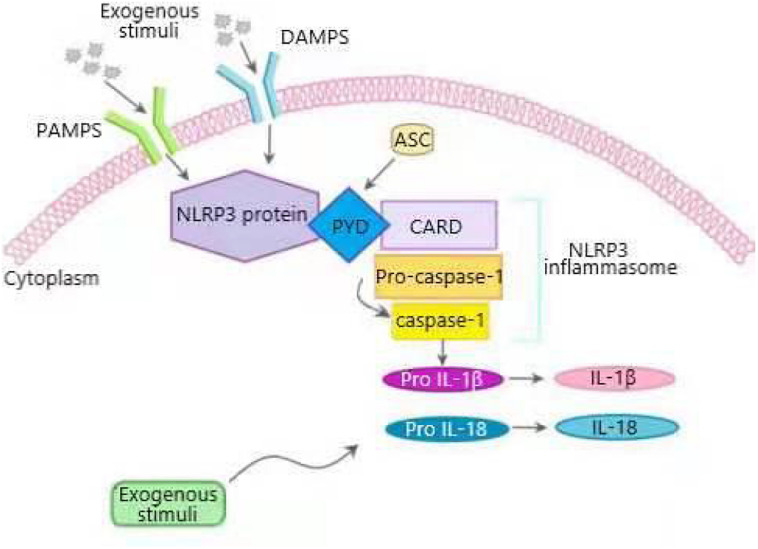
Overview of NLRP3 inflammasome activation process. NLRP3 inflammasome is composed of sensor NLRP3 protein, adapter apoptosis related spot like protein (ASC) containing N-terminal PYRIN-PAAD-DAPIN domain (PYD), C-terminal caspase recruitment domain (CARD), and effector pro-caspase-1. When cells are stimulated by pathogen related molecular patterns (PAMPS) and damage related molecular patterns (DAMPS), NLRP3 inflammasome is activated. Activated NLRP3 interacts with ASC through PYD domain, and pro-caspase-1 binds to ASC through CARD to form a large cytoplasmic complex, thus activating caspase-1. Active caspase-1 cleaves proinflammatory cytokines interleukin-1 β (IL-1β), and IL-18 from their precursors into bioactive forms that induce inflammation.

Inflammasome, a complex composed of many proteins, is an important part of the innate immune system and used to detect the presence of the infection, the pathogens and the metabolic alarms in cells (Elliott and Sutterwala, 2015; Jo et al., 2016). Five major inflammasomes have been identified: NLRP1, NLRC4, RIG-I, AIM2 and NLRP3. These inflammasomes consist of inflammasome adaptor protein, an active NLRP receptor, caspase-1, and apoptosis-associated speck-like protein containing CARD (ASC) (Barbe et al., 2014). NLRP3 inflammasome is the most thoroughly studied inflammasome which is mainly expressed in myeloid cells such as macrophages (O’connor et al., 2003). When the host is stimulated by the exogenous or endogenous stimuli, NLRP3 is activated to lead to the recruitment of pro-caspase-1 and ASC in macrophages. Stimulated NLRP3 interacts with pro-caspase-1 and ASC to form a large cytoplasmic complex, thus activating caspase-1. Actived caspase-1 cleaves pro-IL-1β and pro-IL-18 into IL-1β and IL-18, which induces inflammation by promoting the production of chemokines, proinflammatory cytokines and growth factors (Prochnicki et al., 2016; Figure 2). NLRP3 inflammasome was activated in two steps. The first signals (signal 1) indicating infection or tissue damage, including toll like receptor 4, a pattern recognition receptor capable of recognizing lipopolysaccharide (LPS) and a series of endogenous risk signals, activate the inflammatory transcription factor NF-κβ and increase the expression of NLRP3, pro-IL-1β, and pro-IL-18. Then, the second activation signal (signal 2) indicating cell damage, including extracellular adenosine triphosphate (ATP), urate and cholesterol crystal, induces the assembly of inflammasome and the autolysis of procaspase-1, activates caspase-1, and cleaves pro-IL-1β and pro-IL-18 into their active forms (Sokolova et al., 2019). Abnormal activation of NLRP3 inflammasome can lead to a variety of diseases including metabolic diseases (Meyers and Zhu, 2020).

## The Interplay Between Autophagy and NLRP3 Inflammasome in Glucose and Lipid Metabolic Disorders

### The Interplay Between Autophagy and NLRP3 Inflammasome in Impaired Wound Healing

In recent years, the prevalence of diabetes has increased significantly and become an important public health problem in the world (Balakumar et al., 2016). An important characteristic of diabetes is impaired wound healing (Falanga, 2005). It has been reported that NLRP3 inflammasome-mediated inflammatory response in macrophage is one of the key factors of impaired wound healing in diabetic mice (Bitto et al., 2014). Dai et al. (2017) found that high glucose (HG) activated NLRP3 inflammasome, increased the level of IL-1β and ROS production, and suppressed autophagy by decreasing the protein expression of LC3-II and increasing the protein expression of p62 in macrophages from human diabetic wound. Pre-treatment with resveratrol (an autophagy agonist) counteracted the effects of HG, suggesting that autophagy mediated the HG-induced NLRP3 inflammasome and the ROS production was involved in autophagy-associated NLRP3 inflammasome activation. Moreover, the application of ROS scavenger NAC notably inhibited HG-induced NLRP3 inflammasome, indicating that enhancing autophagy supressed HG-induced NLRP3 inflammasome through reducing ROS production. Autophagy can selectively remove the damaged mitochondria from which ROS mainly comes (Ding et al., 2014; Yang et al., 2014), therefore, it can be inferred that autophagy can inhibit ROS-mediated NLRP3 inflammasome activation through clearing damaged mitochondria, which suggests a new treatment strategy for diabetic wound healing. The relationship among autophagy, ROS and NLRP3 inflammasome needs further study.

### The Interplay Between Autophagy and NLRP3 Inflammasome in Diabetic Nephropathy

Consistent with the above, ROS-mediated NLRP3 inflammasome activation is also involved in diabetic nephropathy (DN). DN is one of the major microvascular complications of diabetes, and has become a common cause of end-stage renal disease (Gao et al., 2016). Many studies have shown that autophagy and inflammation play a key role in the pathological process of DN (Kitada et al., 2011; Feng et al., 2016). Gao et al. (2019) found that in glomerular mesangial cells stimulated by HG, the levels of NLRP3 inflammasome, IL-1β and ROS were increased, indicating that ROS-mediated NLRP3 inflammasome was activated to promote the development of DN. HG promoted autophagy in a short period (<12 h), but inhibited autophagy in a long period (72 h). Meanwhile, HG increased the level of ROS, NLRP3 inflammasome and IL-1β in a time-dependent manner, moreover, treatment cells with rapamycin (a specific activator of autophagy) for 72 h decreased the levels of NLRP3 inflammasome, IL-1β and ROS, suggesting that HG-regulated autophagy negatively regulated ROS-mediated NLRP3 inflammasome. In summary, in a short period of treatment, HG-induced autophagy inhibits ROS-mediated NLRP3 inflammasome and improve DN, while a long period of treatment, HG inhibits autophagy to activates ROS-mediated NLRP3 inflammasom to aggravate DN. The reason may be that HG causes mitochondrial damage, which induces autophagy to clear the damaged mitochondria-derived ROS. In a short period of time, the inhibition of HG on autophagy was less than the induction of ROS on autophagy, so the level of autophagy increased. With the extension of time, the induction of autophagy weakens, while high glucose inhibits autophagy to activate ROS-mediated NLRP3 inflammasome.

In addition to the above glomerular mesangial cell inflammation, NLRP3 inflammasome-mediated inflammation of tubulointerstitial also contributes to the development of DN (Chen et al., 2013; Hwang et al., 2017). Optineurin (OPTN), initially considered to be a regulator of NF-κβ signaling, is an important regulator of mitophagy in patients with DN, and reducing OPTN expression leads to premature senescence of renal tubular cells to promote DN (Slowicka et al., 2016; Chen et al., 2018). The results of Chen et al. (2019) showed that the expression of optineurin (OPTN) was reduced and correlated negatively with NLRP3 inflammasome activation in patients with DN. In primary mouse renal tubular epithelial cells (RTECs) treated with HG, the expression level of OPTN significantly decreased, the level of NLRP3 expression, cleaved caspase-1, and IL-1β, and the release of IL-1β increased. Moreover, the expression of mitochondrial fission protein Drp1 increased, and the expression of mitochondrial fusion protein Mitofusin 2 and mitochondrial membrane potential decreased, indicating that HG inhibited OPTN, activated NLRP3 inflammasome-mediated inflammation and promoted mitochondrial dysfunction. While overexpression of OPTN in RTECs counteracted the HG effects on OPTN and NLRP3 inflammasome-mediated inflammation, and silencing of OPTN had the opposite result, suggesting that OPTN inhibited HG-induced activation of NLRP3 inflammasome. HG impaired mitophagy by decreasing the level of LC3II in RTECs. The mitophagy-specific inhibitor (Mdivi-1) could activate NLRP3 inflammasome in the presence of HG, while the autophagy agonist (Torin) had the opposite effect, indicating that mitophagy supressed NLRP3 inflammasome activation induced by HG. In addition, overexpression of OPTN notably increased mitophagy and silencing of OPTN induces mtROS production and activation of NLRP3 inflammasome. Collectively, it can be inferred that OPTN inhibited HG-induced NLRP3 inflammasome by enhancing mitophagy-mediated clearance of ROS. The mechanism of OPTN regulating mitophagy, especially the signal pathway, needs to be further elucidated.

On the contrary to autophagy-mediated regulation of the NLRP3 inflammasome, NLRP3 inflammasome also can regulate autophagy. In podocytes of DN mice induced by high-fat diet/streptozotocin (HFD/STZ), the number of autophagosomes decreased and the level of NLRP3 inflammasome expression and renal proinflammatory cytokines such as IL-1β increased, meanwhile, the similar results were obtained in human DN biopsies, suggesting that podocyte autophagy was inhibited and NLRP3 inflammasome was activated. In podocyte, with the activation of NLRP3 inflammasome by LPS plus ATP, the ratio of LC3II/I, the expression level of beclin 1 and nephrin (which meant podocyte injury), and the formation of autophagosomes decreased, indicating that podocyte autophagy is inhibited by the activation of NLRP3 inflammasome to aggravate the damage of podocyte. While silencing NLRP3 by siRNA had the opposite results. Therefore, appropriate changes of autophagy and inflammasome may potentially improve DN (Hou et al., 2020). The specific mechanism of NLRP3 negatively regulating autophagy needs to be further explored.

### The Interplay Between Autophagy and NLRP3 Inflammasome in Diabetic Cardiomyopathy

Diabetic cardiomyopathy (DCM) is an important complication of diabetes and associated with inflammation, oxidative stress, impaired calcium homeostasis and mitochondrial damage (Huynh et al., 2014; Kobayashi and Liang, 2015). Autophagy and NLRP3 inflammasome have been reported to be involved in the pathological process of DCM (Xie et al., 2011; Luo et al., 2014). Visceral adipose tissue derived serine protease inhibitor (vaspin), firstly discovered in 2005, is an adipokine, which has significant anti-inflammatory effect (Li et al., 2008). In STZ-induced diabetes rat model, Vaspin improved cardiac function, cardiomyocyte apoptosis, myocardial tissue morphology and mitochondrial morphology to alleviate DCM. Mechanistic studies of the above effects revealed that LC3II/I ratio decreased and p62 expression increased in diabetic hearts, while Vaspin treatment abrogated the change to promote autophagy. Moreover, Vaspin also suppressed NLRP3 inflammasome through reducing the expression level of NLRP3 inflammasome and IL-1β. In HG-induced H9C2 cells, treatment with the autophagy inhibitor (3-MA) abolished the inhibitory effects of Vaspin on NLRP3 inflammasome, indicating that Vaspin alleviated STZ-induced myocardial injury by suppressing NLRP3 inflammasome activation via promoting autophagy. In addition, Vaspin inhibits ROS production and mitochondrial membrane depolarization in HG-induced H9C2 cells (Li et al., 2019). It can be inferred that there may be two mechanisms of myocardial protection in the above process: one is that Vaspin-induced autophagy can clear the damaged mitochondria to reduce the production of ROS to inhibit NLRP3 inflammasome; another is Vaspin improve damaged mitochondria to reduce the production of ROS to inhibit NLRP3 inflammasome.

### The Interplay Between Autophagy and NLRP3 Inflammasome in Obesity-Induced Insulin Resistance

Obesity causes chronic low-grade inflammation, leading to insulin resistance, which is characteristic of type 2 diabetes mellitus (Xu et al., 2003; Arkan et al., 2005; Shoelson et al., 2007). It has been reported that NLRP3 inflammasome perceives obesity related risk signals and participate in inflammation and insulin resistance caused by obesity (Vandanmagsar et al., 2011). Berberine (BBR) is a natural plant product and beneficial to diabetes and dyslipidemia (Kong et al., 2004; Turner et al., 2008). Zhou et al. (2017) found that BBR could significantly inhibit the activation of NLRP3 inflammasome and the release of IL-1β induced by saturated fatty acid palmitate (PA) in bone marrow-derived macrophages (BMDMs). Moreover, it also significantly increased the autophagy level and decreased mitochondrial ROS production in LPS plus PA-treated macrophages, while Beclin1 (an autophagy marker gene) knockout and 3-MA reversed the inhibitory effect of BBR on NLRP3 inflammasome and mitochondrial ROS production, indicating that BBR suppressed NLRP3 inflammasome-mediated inflammation and mitochondrial ROS production through enhancing autophagy. In addition, BBR could increase phosphorylation of AMPK (adenosine activated protein kinase) and caused a decrease of mammalian target of rapamycin (mTOR) in LPS plus PA-treated BMDMs, AMPK inhibitor, Ara-A, blocked most of the effects of BBR, suggesting that AMPK signal may be involved in BBR-induced autophagy. The similar effects of BBR were obtained in a HFD induced insulin resistance model. In conclusion, BBR inhibit obesity-induced inflammation and insulin resistance by activating AMPK dependent autophagy through suppressing mitochondrial ROS production in adipose tissue macrophages, which needs to be further studied. Targeting the effect of autophagy on NLRP3 with oral small molecule compound BBR might improve insulin resistance. Similar to the above results, our previous studies have shown that enhancing autophagy by hydrogen sulfide (H_2_S) suppresses NLRP3 inflammasome through AMPK-mTOR pathway in L02 cells induced by oleic acid (Wang et al., 2019). AMPK-mTOR pathway is an important signaling pathway for the interaction between autophagy and NLRP3 inflammasome.

### The Interplay Between Autophagy and NLRP3 Inflammasome in High Fat and High Sugar Diets-Induced Heart Damage

High energy diets rich in fat and *high sugar* leads to low-grade systemic inflammatory response and increases the risk of cardiovascular disease (CVD) (Minihane et al., 2015). NLRP3 inflammasome-mediated inflammation plays an important role in the cardiomyopathy of rats treated with HFD and STZ (Luo et al., 2014). Genetic ablation of NLRP3 or inhibition of NLRP3 by MCC950 could relieve high-sugar diet (HSD), HFD or high sugar/fat diet induced obesity, cardiomyocyte apoptosis and inflammation, and improve the antioxidant capacity to ameliorate cardiac injury. Mechanistic studies revealed that genetic ablation of NLRP3 or inhibition of NLRP3 by MCC950 could induce autophagy by increasing LC3-II level and decreasing p62 level (Pavillard et al., 2017). HFD-induced obesity can impair autophagy in cardiomyocytes (He et al., 2012), while the inhibition of NLRP3-inflammasome and promotion of autophagy can improve HFD-induced cardiac injury (Abderrazak et al., 2015). Collectively, it can be inferred that inhibition of NLRP3-inflammasome improve HSD, HFD, or high sugar/fat diet induced heart damage through promoting autophagy, which needs to be further explored. It is predicted that therapeutic targetting of autophagy/NLRP3 inflammasomes can improve obesogenic diet-induced heart injury.

## The Interplay Between Autophagy and NLRP3 Inflammasome in Uric Acid Metabolism Disorders

Uric acid nephropathy (UAN) is one of the most common metabolic diseases, which leads to kidney damage (Wu et al., 2012; Hou et al., 2014). Urate can activate NLRP3 inflammasome and NLRP3 inflammasome-mediated inflammation is involved in kidney injury (Wang et al., 2012). Hu et al. (2018) found that uric acid activated NLRP3 inflammasome, promoted caspase-1 activation, and induced renal inflammation, leading to the generation and development of UAN. Weicao, a traditional Chinese medicine preparation, could reduce uric acid, improve proteinuria and renal insufficiency and reduce renal tissue crystals to avoid renal interstitial fibrosis. It also reduced the levels of NLRP3 inflammasome, IL-18 and IIL-1β, and significantly increased the levels of beclin-1, LC3-II, and LC3-II/LC3-I ratio, suggesting that Weicao inhibited NLRP3 inflammasome-mediated inflammation and promoted autophagy to improve UAN. In addition, autophagy inhibitor 3-MA abolished the effects of Weicao on NLRP3 inflammasome, suggesting autophagy mediated the inhibitory effect of Weicao on NLRP3 inflammasome activation. NLRP3 inflammasome activator, ATP, blocked the effects of Weicao capsule on autophagy, indicating that NLRP3 inflammasome participated in Weicao induction of autophagy. The similar results were obtained *in vitro*. Collectively, Weicao could ameliorate renal injury of UAV rats through inhibiting NLRP3 inflammasome and inducing autophagy. It is predicted that therapeutic targetting of autophagy/NLRP3 inflammasomes can ameliorate UAN. The relationship between autophagy and NLRP3 inflammasome is very complex, which needs to be further studied.

## The Interplay Between Autophagy and NLRP3 Inflammasome in Other Types of Metabolic Disorders

### The Interplay Between Autophagy and Nlrp3 Inflammasome in Aging Related Metabolic Disorders

Endothelial cell senescence is an important factor in the pathogenesis of senile vascular diseases, which has been observed in diabetic CVDs (Yokoi et al., 2006). Studies have shown that autophagy is closely related to the accelerated aging of vascular endothelial cells, and enhanced autophagy may have a strong anti-aging effect (Wohlgemuth et al., 2014; Lee et al., 2015). Purple sweet potato pigment (PSPC), a flavonoid compound isolated from purple sweet potato, can inhibit the aging of endothelial cells of diabetic patients (Sun et al., 2015). Sun et al. (2019) found that PSPC promoted autophagy by increasing LC3 II and decreasing p62 level and suppressed the premature senescence of endothelial cells induced by HG. The similar results were obtained in diabetic mice *in vivo*. Rapamycin, an autophagy promotor, inhibited HG-induced endothelial cell senescence, while 3-MA accelerated endothelial cell senescence, indicating that autophagy mediated PSPC suppression of endothelial cell senescence. Moreover, PSPC and Rapamycin suppressed HG-induced NLRP3 inflammasome, and 3-MA abolished the inhibitory effect of PSPC on NLRP3 inflammasome, suggesting that PSPC suppressed NLRP3 inflammasome by promoting autophagy. P62, an autophagic receptor, acts as a molecular bridge to deliver substrates to autophagosomes (Seibenhener et al., 2004). P62 binded to NLRP3 inflammasome and transported it to lysosome for degradation. In the senescent endothelium of diabetic mice, HG impaired autophagy to inhibit P62-NLRP3 inflammasome degradation in lysosome, while PSPC had the opposite effects, which was the mechanism that PSPC inhibits NLRP3 inflammasome by promoting autophagy (Sun et al., 2019).

Nucleotide-binding oligomerization domain-like receptor family, pyrin domain-containing 3 inflammasome-mediated inflammation disorder is closely related to aging-related metabolic disorders (Finkel, 2015; Cordero et al., 2018). Marin-Aguilar et al. (2020) found that MCC950, a specific NLRP3 inhibitor, could reduce body weight, increase insulin sensitivity, improve hepatic metabolic dysfunction by reducing hepatic transaminases, lactate dehydrogenase, and creatine phosphokinase and alleviate steatosis and fibrosis in aged mice. Mechanistic studies revealed that MCC950 inhibited PI3K/Akt/mTOR pathway and enhanced autophagy by increasing LC3-II level and decreasing p62 level to improve the metabolism and liver dysfunction of old mice *in vivo* and *in vitro*. It can be inferred that NLRP3 inflammasome inhibition with MCC950 improves metabolism disorder via promoting autophagy through inhibiting PI3K/Akt/mTOR pathway in aged mice, which needs to be further studied by using autophagy inhibitors and the activator of PI3K/Akt/mTOR pathway.

### The Interplay Between Autophagy and NLRP3 Inflammasome in Inflammation-Related Metabolic Disorders

Evidence indicates that mild chronic inflammation, including adipose tissue inflammation, contributes to metabolic disorders and is associated with many metabolic diseases, such as type 2 diabetes mellitus, insulin resistance and obesity (Weisberg et al., 2003; Lumeng and Saltiel, 2011; Harte et al., 2013). IL-1β activated by NLRP3 inflammasome can worse insulin resistance in human adipocytes (Gao et al., 2014), moreover, inhibition of IL-1β can reduce hyperglycemia in obese/diabetic rats (Ehses et al., 2009). So, inhibition of NLRP3 inflammasome-mediated production of IL-1β can improve the metabolic disorder associated with adipose tissue inflammation. AEDC is a cycloartane triterpenoid isolated from the whole plant of *Amanita vaginata* (Fang et al., 2019). Zhang et al. (2020) found that AEDC suppressed LPS plus ATP-induced NLRP3 inflammasome activation and IL-1β secretion in THP-1 macrophages. Moreover, LPS plus ATP notably decreased the protein levels of Atg5, Atg7, beclin 1 and the ratio of LC3-II/LC3-I and increased the level of p62, while AEDC had opposite effects, indicating that AEDC restored autophagy impaired by LPS plus ATP. 3-MA abolished the AEDC suppression of NLRP3 inflammasome activation induced by LPS plus ATP, suggesting that AEDC inhibited LPS plus ATP-induced NLRP3 inflammasome activation by promoting autophagy. Mechanistic studies showed that AEDC increased the expression level of NAD^+^-dependent deacetylase sirtuin-3 (SIRT3) deacetylase and enhanced its deacetylation activity to restore mitochondrial dysfunction induced by LPS plus ATP. 3-TYP, a selective SIRT3 inhibitor, reversed AEDC effect on autophagy, indicating that AEDC inhibited NLRP3 inflammasome activation to ameliorate inflammation-related metabolic disorders by promoting autophagy via SIRT3. Here, autophagy still suppresses NLRP3 inflammasome by clearing damaging mitochondria derived ROS.

## Conclusion

**FIGURE 3:**
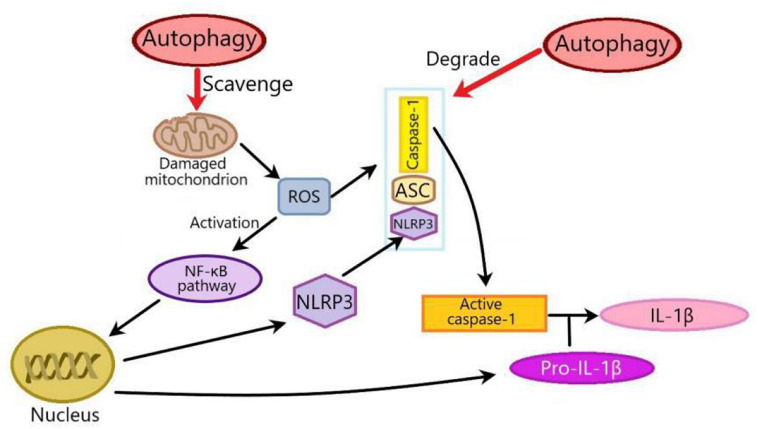
Two mechanisms of the effects of autophagy on NLRP3 inflammasome in metabolic disorders. There are two mechanisms of the effects of autophagy on NLRP3 inflammasome: one is that autophagy inhibits NLRP3 inflammasome by scavenging ROS from damaged mitochondria. Mitochondria-derived ROS can activate NF-κβ pathway to promote the transcription of NLRP3 and pro-IL-1β, thus activating NLRP3 inflammasome. The other is that autophagy inhibits NLRP3 inflammasome through degrading inflammasome components such as pro-IL-1β, NLRP3, caspase-1, or ASC. ROS, Reactive oxygen species; NF-κβ, nuclear factor kappa-B; ASC, apoptosis-associated speck-like protein.

The interplay between autophagy and NLRP3 inflammasome plays an important role in metabolic disorders. In this review, there are two mechanisms of the effects of autophagy on NLRP3 inflammasome in metabolic disorders: One is that autophagy suppresses NLRP3 inflammasome by inhibiting ROS production through scavenging damaged mitochondria. ROS released from mitochondria can activate the NF-κβ pathway to promote the transcription of NLRP3 and pro-IL-1β, thus activating NLRP3 inflammasome. The other is that autophagy can isolate and promote the degradation of inflammasome components such as pro-IL-1β, NLRP3, caspase-1, and ASC (Figure 3). Generally speaking, in addition to the above two ways, autophagy also inhibits NLRP3 inflammasome by phosphorylating NLRP3 (Cao et al., 2019). Whether autophagy can influence NLRP3 inflammasome by phosphorylating NLRP3 in metabolic disorders remains to be clarified.

Although the interplay between autophagy and NLRP3 inflammasome has potential therapeutic value in metabolic disorders, its mechanism has not been fully explained. For example, in metabolic disorders, does autophagy promote the activation of the NLRP3 inflammasome? What is the mechanism of NLRP3 inflammasome acting on autophagy? And are there any side effects of targeting autophagy/NLRP3 inflammasome to improve metabolic disorder? With the deepening of the research, targeting autophagy/NLRP3 inflammasome will provide a new method for the treatment of metabolic disorders related diseases.

## Author Contributions

HW devised, wrote, and funded the review. SL wrote and funded the review. XL drew the pictures of the review.

## Conflict of Interest

The authors declare that the research was conducted in the absence of any commercial or financial relationships that could be construed as a potential conflict of interest.
